# Methylation of cancer related genes in tumor and peripheral blood DNA from the same breast cancer patient as two independent events

**DOI:** 10.1186/1746-1596-6-116

**Published:** 2011-11-30

**Authors:** Tomasz K Wojdacz, Britta B Thestrup, Jens Overgaard, Lise Lotte Hansen

**Affiliations:** 1 The Lundbeck Foundation Centre for International Research in Radiation Oncology, Nørrebrogade 44, Building no. 5, Aarhus University Hospital, DK-8000 Aarhus C, Denmark; 2 Department of Experimental Clinical Oncology, Aarhus University Hospital, Nørrebrogade 44, Building no. 5, DK-8000 Aarhus C, Denmark; 3 Department of Biomedicine, University of Aarhus, Wilhelm Meyers Allé 4, DK-8000 Aarhus C, Denmark

**Keywords:** Methylation, cancer predisposition, *BRCA1*, *APC*, *RASSF1A*.

## Abstract

**Background:**

Recently it has been suggested that acquisition of methylation of the *BRCA1 *promoter detectable in peripheral blood (PB) DNA, could give raise to development of breast cancer. In this study, we aimed to investigate a relationship between methylation of three breast cancer related genes in PB DNA, and tumor specific (somatic) methylation of these genes in the same individual.

**Findings:**

We have examined methylation status of the *BRCA1, APC *and *RASSF1A *promoter regions in a panel of 75 breast tumor and PB DNA samples from the same individual. In our study group, 4.0% of the patients displayed methylation of *BRCA1 *and *APC *in both tumor and the corresponding PB DNA. At the same time despite of marked methylation in tumor DNA, no methylation of *BRCA1 *and *APC *was seen in PB DNA of 4.3% and 2.7% of the patients respectively. The *RASSF1A *promoter did not show methylation in PB DNA.

**Conclusions:**

Our results show that for at least a subset of cancer patients methylation of certain cancer related genes in PB DNA does not seem to be directly linked to somatic methylation of the same genes in tumor DNA, and therefore may only be specific to PB DNA.

## 

The mechanism of methylation dependent gene deactivation and its significance to cancer pathogenesis is well described, with hypermethylation of tumor suppressor genes, affecting transcriptional activity of the genes, considered to be one of the most important drivers of carcinogenesis. Recently, much attention is paid to the phenomenon of hypermethylation of disease related genes in peripheral blood (PB) DNA and its involvement in the pathology of cancer and other diseases [1-6]. The origins of this phenomenon are unknown. However, it can be hypothesized that aberrant methylation of genes in PB DNA may be a consequence of germ line transmitted methylation changes or somatic aberrations that occurred during early development or through life time under specific environmental conditions. Transmission of methylation changes through germ line is still a problematic notion. There are only limited evidence showing that methylation of certain genes e.g. *MLH1 *in some cases can be passed through germ line in non-Mendelian fashion [7-11]. Recently, two studies have shown that paternal diet can have an influence on the methylation pattern of the offspring [12,13]. This further supports the significance of germ line transmission of methylation changes, however, these findings have to be more extensively researched in the future. As for environmental pressure on the individuals methylome, the influence of different chemicals on the somatic methylation pattern of the exposed subjects has been demonstrated in animal models, and proven to be especially damaging when the exposure occurred in the early stages of development (as reviewed in [14]). In humans there is mounting epidemiological evidence that environmental exposure can predispose to adult onset diseases. However it is still not clear, how the interactions between individual organisms and the environment occur, and to what extent they involve methylation changes.

Disregarding the origin, the intra individual methylation differences in human PB DNA are being increasingly reported in the literature [4-6]. Furthermore, these changes have been suggested to be a part of a disease predisposition mechanism, which could be based on the theory of constitutional methylation [2].

Constitutional gene methylation was initially defined as abnormal gene methylation observed in all tissues of the body [15]. Constitutional methylation is most likely affecting genes in a mono allelic fashion and if acquired during development, it can be distributed to all tissues of the organism in a mosaic pattern (and therefore seen at very low levels in affected tissues) [2,4]. Drawing analogy from somatic methylation in cancer, constitutional mono allelic methylation changes are likely to render the affected individual prone to development of neoplastic (and other) diseases. This is due to the fact that only one additional hit would be required (according to Knudson's hypothesis of tumor suppressor deactivation [16]) to abolish expression of the constitutionally mono allelic methylated gene and initiate or contribute to carcinogenesis. Moreover, allelic insufficiency could also be a disease-initiating factor.

In the first report, suggesting a link between methylation of the *BRCA1 *promoter in PB DNA and development of breast cancer with methylated *BRCA1*, the authors examined methylation status of the *BRCA1 *promoter in tumors and PB DNA from three breast cancer patients [3]. They showed that the *BRCA1 *promoter was methylated in both PB DNA samples and matched tumor DNA in all examined individuals [3]. The observed methylation was never seen at a level suggesting monoallelic specificity (50% methylation) but only at low levels (5-14%) reflecting high degree of mosaicism in the screened cell populations. Despite the promising results of this study showing a putative elegant mechanism of direct contribution of constitutional methylation to carcinogenesis, two follow up studies did not result in similar conclusions. In those studies the authors have examined methylation status of the *BRCA1 *promoter in PB and paired tumor DNA, however only a subset of the tumors, developed by patients with PB *BRCA1 *methylation, harbored tumor specific methylation of *BRCA1 *[2,17]. The study by Wong et al. [2] involved 12 breast cancer patients with paired tumor and PB DNA samples. The authors in this study reported generally low levels of methylation detected in PB (for most of the samples less than 5%) and furthermore, three patients with *BRCA1 *methylation in PB DNA did not display *BRCA1 *methylation in the paired tumor sample. A subsequent study by Iwamoto et al. [17] showed a more significant lack of direct correlation between methylation of *BRCA1 *in PB DNA and matched tumor samples.

Based upon the above results we aimed to investigate the presence or absence of a correlation between DNA methylation of cancer related genes in PB and paired tumor DNA from breast cancer patients.

We hypothesized that if the observed methylation of genes in PB DNA reflects constitutional methylation, the same methylation pattern has to be present in tumor DNA (as it originates from healthy tissue harboring constitutional methylation of the specific gene). Consequently if the methylation of those genes cannot be found in tumor DNA, the methylation observed in PB DNA is only specific to PB DNA (not constitutional) and does not directly contribute (in this case) to breast carcinogenesis of the affected individual.

In our study methylation of the *BRCA1, APC *and *RASSF1A *promoter regions was analyzed in 75 paired breast tumor and PB DNA samples using the MS-HRM (Methylation Sensitive High Resolution Melting) protocol. All MS-HRM assays were designed according to the guidelines published in [18,19]. The primer sequences used have been published in [20]. The regions targeted by the assays, spanning promoters of the screened genes are shown in Table 1. The experiments were performed as previously described [21]. Briefly, tumor DNA was extracted as described in [22], and a modified salting-out protocol was used for purification of DNA from peripheral blood [23]. 100 ng of genomic DNA was bisulfite modified using EpiTect Bisulfite Kit (Qiagen). The LighCycler^® ^480 platform (Roche) was used for both PCR amplifications and the subsequent HRM analyses. The PCR mixes consisted of 1× LightCycler^® ^480 HRM Master mix Roche, 3 mM Mg^+2^, 0.5 μM of each primer and 4 ng (theoretically) of bisulfite modified DNA template. All reactions were run in triplicates. The methylation status of each sample was scored by comparison of the HRM profile of the sample to the HRM profiles obtained from dilutions of the methylated bisulfite modified template in an unmethylated background (Millipore). The data in this study were analyzed as previously described [21], where MS-HRM and Sanger sequencing were used to confirm that any aberrations of the HRM profile from the profile of the PCR product amplified from 0% methylation template are positive for methylation. Due to the fact that all published studies report the constitutional methylation to occur at very low levels (mosaic fashion) instead of the expected 50% methylation level that could be anticipated for mono allelic methylation, our data analysis was similar to previously published results based on qualitative methylation assessment. Nevertheless, MS-HRM data allow for quantitative methylation measurement and the Figures 1, 2 and 3 depict the representative MS-HRM results. It can be argued that for the patients displaying the same methylation pattern in tumor and PB DNA, the detectable in PB DNA methylation, is not PB specific, but derived from circulating tumor cells and/or free circulating tumor DNA. However, it was previously shown that detection of tumor circulating cells is only possible when using enrichment technologies [24], and moreover our peripheral blood sample processing protocol dilutes free circulating tumor DNA and tumor cells under detection limit of MS-HRM (as described in [2]).

**Table 1:** Frequencies of methylation of analyzed genes in PB and breast tumors.

GENE	Chromosomal region targeted by assay *	Number of paired samples used/excluded**	Methylated in both blood and tumor Sample no. (%)	Methylated only tumor***	Methylated only PBL Sample no. (%)
*BRCA1*	chr17:41, 277, 382-41, 277, 409	75/6	3 (4.3%)	23%	3 (4.3%)

*APC*	chr5:112, 073, 470-112, 073, 516	75/3	3 (4.1%)	83%	2 (2.7%)

*RASSF1A*	chr3:50, 377, 755-50, 378, 089	75/2	0%	97%	0

* UCSC Genome Browser on Human Feb. 2009 (GRCh37/hg19) Assembly

** Samples excluded due to failure of PCR amplification

*** Out of all tumor samples used

**Figure 1:**
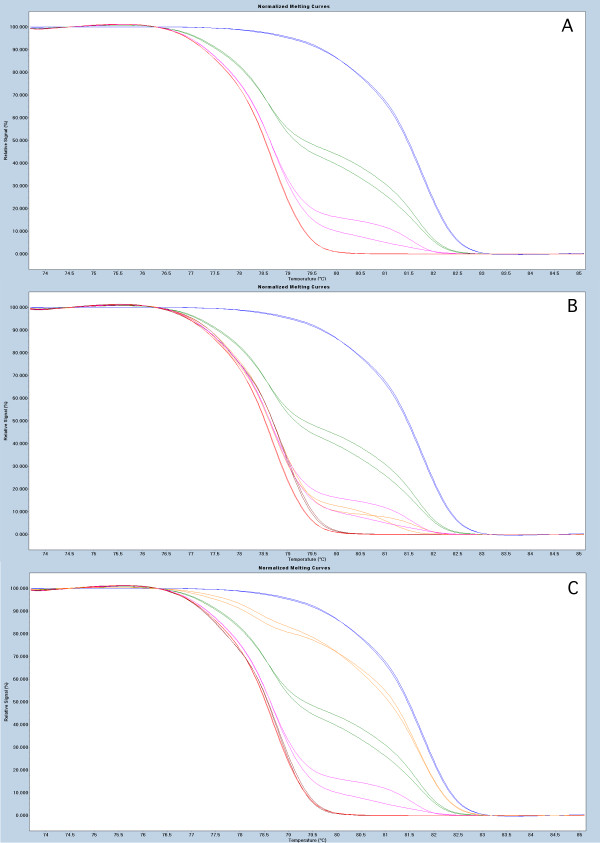
**Representative results for MS-HRM based screening for *APC *methylation**. Panel 1A, show the sensitivity of the assays with MS-HRM profile characteristic for 100% - blue, 10% - green, 1%- pink and 0% - red, mixes of methylated template in unmethylated background. Panel 1B, examples of PB sample positive (orange) and negative (brown) for methylation. Panel 1C, examples of the tumor sample positive (orange) and negative (brown) for methylation.

**Figure 2:**
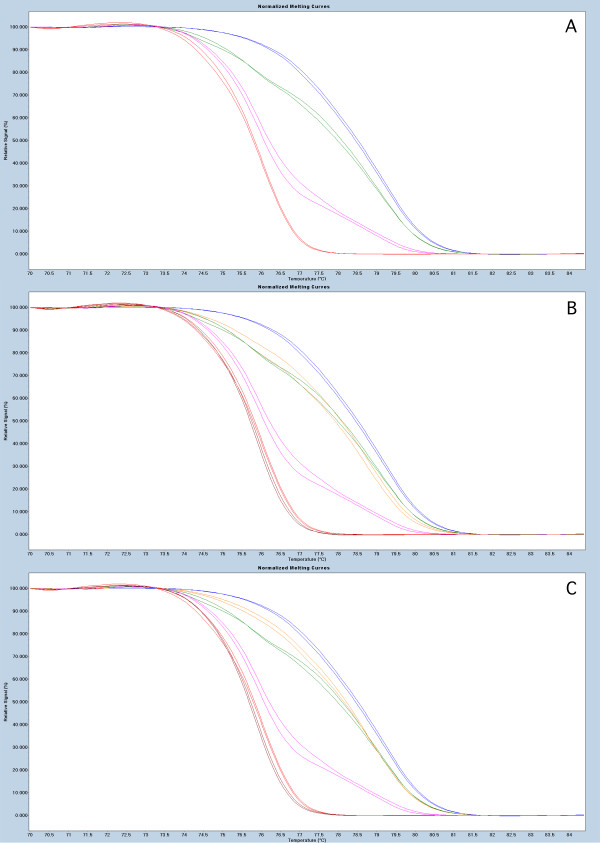
**Representative results for MS-HRM based screening for *BRCA1 *methylation**. Panel 1A, show the sensitivity of the assays with MS-HRM profile characteristic for 100% - blue, 10% - green, 1%- pink and 0% - red, mixes of methylated template in unmethylated background. Panel 1B, examples of PB sample positive (orange) and negative (brown) for methylation. Panel 1C, examples of the tumor sample positive (orange) and negative (brown) for methylation.

**Figure 3:**
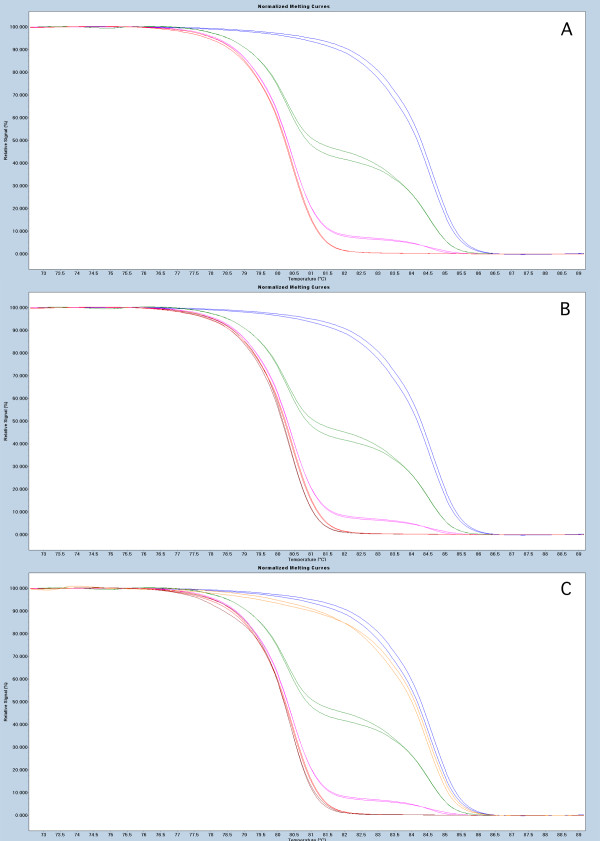
**Representative results for MS-HRM based screening for *RASSF1A *methylation**. Panel 1A, show the sensitivity of the assays with MS-HRM profile characteristic for 100% - blue, 10% - green, 1%- pink and 0% - red mixes of methylated template in unmethylated background. Panel 1B, example of PB sample positive (orange) for methylation. Panel 1C, examples of the tumor sample positive (orange) and negative (brown) for methylation.

All three genes analyzed in our study showed methylation in the tumor samples suggesting as previously reported, their involvement in breast cancer pathogenesis Table 1. We observed high frequencies of methylation for all three genes, however the methylation frequencies for *APC *and *RASSF1A *were higher than reported in the literature http://www.pubmeth.org. This may be attributed either to an exceptionally high methylation prevalence in our samples or more likely to a high sensitivity of the MS-HRM technology [25].

Two of the examined genes, *BRCA1 *and *APC*, showed methylation in both tumor and paired PB DNA at frequencies of 4.4% and 4.1%, respectively (see Table 1 for details) with one of the paired samples showing methylation of both *APC *and *BRCA1 *in tumor and PB DNA. However, at the same time three of the samples in our panel did not show methylation of *BRCA1 *in tumor DNA despite marked methylation in PB DNA. The same was seen for *APC *in two of the samples. *RASSF1A *did not show methylation in any of the PB samples.

Our study shows that a direct link between methylation of cancer related genes in PB DNA and development of cancer is questionable. If a direct link existed and as previously suggested detectable in PB DNA hypermethylation was constitutional [2], all patients with PB DNA methylation should display the same specific methylation pattern in the paired tumor. This is due to the fact that tumor DNA originates from healthy tissue, which (similar to blood tissue) should harbor constitutional methylation that in turn would predispose the affected individual to cancer development. The fact that we have not seen the same methylation pattern for a subset of our paired samples suggests independence of the methylation events in PB DNA and during tumor development. However, at the same time basing on current results, we cannot rule out the presence of a direct link between those two events for the subset of patients displaying methylation in both PB and tumor DNA.

In conclusion, the fact that methylation of the *BRCA1 *gene in PB DNA correlates with increased risk of breast cancer, allows to anticipate that aberrant methylation of genes in PB and disease predisposition are linked. Especially considering the study by Iwamoto et al. and the study by Wong at al. both indicating a strong correlation between methylation of *BRCA1 *in PB and breast cancer incidence. However, our present and previously published results do not confirm that the mechanism of that interaction is based solely on constitutional methylation and suggests independence of those two events for at least a subset of cancer patients.

## Competing interests

TKW and LLH are listed as inventors on patent pending application on aspects of MS-HRM technology. JO and BBT have no competing interests.

## Authors' contributions

TKW performed the experiments, wrote the manuscript, TBB performed experiments, JO and LLH supervised the experiments wrote and the manuscript. All authors approved the manuscript.

### Financial support

This study was supported by The Danish Cancer Society, CIRRO - The Lundbeck Foundation Center for Interventional Research in Radiation Oncology and The Danish Council for Strategic Research (TKW, JO, LLH), Aase og Ejnar Danielsens Fond and the Toyota Foundation (TKW, LLH, TBB).
